# The Impact of Collaboration Between Science and Religious Education Teachers on Their Understanding and Views of Argumentation

**DOI:** 10.1007/s11165-022-10041-1

**Published:** 2022-02-08

**Authors:** Jessica Chan, Sibel Erduran

**Affiliations:** Department of Education, University of Oxford, 15 Norham Gardens, Oxford, OX2 6PY UK

**Keywords:** Teacher collaboration, Argumentation, Science teaching, Religious education, Interdisciplinarity

## Abstract

Teachers’ understanding and teaching of argumentation is gaining more attention in science education research. However, little is known about how science teachers engage in argumentation with teachers of different subject taking an interdisciplinary perspective that may inspire new pedagogical ideas or strategies. In particular, the positioning of argumentation at the juncture of science and religion is rare. This paper reports an empirical study involving science and religious education (RE) teachers who collaborated on teaching argumentation in three secondary schools in England. Their interdisciplinary collaboration was sustained by a series of professional development sessions over 18 months. Analysis of the interview data unfolds how the teachers’ collaboration impacted their understanding of argumentation and views of teaching their subject. Through working relationally in exploring and teaching argumentation, the science teachers reflected more notable changes than their RE counterparts. Science teachers came to appreciate student voice in the learning process and the role of argumentation in fostering students’ scientific reasoning. The paper is a salient step to researching argumentation in a cross-curricular terrain, particularly in relation to RE. It also sheds light on how collaborating with teachers of another subject bolstered science teachers’ professional development and broke subject barriers.

## Introduction

Argumentation, or the justification of knowledge claims with evidence and reasons (Toulmin, 1958), is a focus in many cross-curricular initiatives (Asterhan & Schwarz, 2016). The process of generating or engaging with arguments and backing up the arguments by evidence and warrant is often framed as *argumentation *(Jiménez-Aleixandre & Erduran, 2007). Research has extensively reported the benefits of argumentation in fostering higher-order thinking skills (Rapanta, 2021). Because of its philosophical root and educational merits, argumentation is inherently a generic skill and is often discussed from a cross-curricular perspective (Joshi, 2016; Staples et al., 2016). Within the subject boundaries in education, argumentation has been widely researched in science (Zohar & Nemet, 2002), literacy (Bloome & Wilkinson, 2012; Lu & Zhang, 2013), language arts (Rapanta, 2021), history (Monte-Sano, 2016), mathematics (Staples & Newton, 2016), and social studies and civic education (Joshi, 2016). Applying argumentation to learning in a range of subjects and disciplines has therefore strengthened its power in and relevance to thinking in interdisciplinary domains.

In this paper, following a review of the literature in related areas, we focus on an empirical study conducted with science and religious education (RE) teachers, who were collaborating about argumentation in the context of a funded research project in England. The three-year project aimed to foster collaboration between the teachers in infusing argumentation in their teaching. The paper illustrates how the teachers’ collaboration on argumentation has impacted their views and understanding of argumentation and its teaching. Although there is research on science teachers’ views about the science and religion comparison (e.g. BouJaoude et al., 2011; Mansour, 2015), very little research has involved both science and RE teachers in the same study on interdisciplinary issues that concern the school subjects of science and religious studies (e.g. McKinney et al., 2014). In England, while there are ample opportunities for argumentation in the RE curriculum (Chan et al., 2021; Godfrey & Erduran, 2021; Ofsted, 2021), the scope in the science curriculum is very limited (Jenkins, 2013; Naylor et al., 2007). Despite these different expectations, this paper intends to contribute to the literature by elucidating how science and RE teachers collaborated to engage their students in argumentation.

## Literature Review

There is extensive research on argumentation in science education (e.g. Duschl & Osborne 2002; Jiménez-Aleixandre & Erduran, 2007; Osborne et al., 2016; Zohar & Nemet, 2002). Increasingly, interdisciplinarity and argumentation are drawing more attention in the education research field for various reasons (Erduran et al., 2019). The emphasis on developing students’ higher-order skills and critical thinking in a cross-curricular context is a vehicle for researching the potentials of argumentation across different subjects. For instance, Crujeiras-Pérez and Jiménez-Aleixandre (2019) proposed that problem-solving in day-to-day life in the twenty-first century requires innovative thinking that adopts an interdisciplinary perspective. We need not wait too long to fully and immensely appreciate the importance of an interdisciplinary approach to argumentation for problem-solving from personal to national and supranational levels. Just months after their claim, the pandemic presented high-stakes and unprecedented challenges to virologists, public health experts, policymakers, healthcare professionals, business owners, teachers, and parents—simply humans from all walks of life in all countries. Advice or new rules swiftly imposed on citizens were communicated as compelling measures backed up by data and warrants. The policies were then scrutinised by a bottom-up rebuttal from citizens evaluating the impacts on the economic, educational, environmental, psychological, and social aspects of life. In short, good decision-making and problem-solving in education matters or others are largely contingent upon interdisciplinarity and argumentation. Developing students for these competencies has become more urgent and profound than most educators had forewarned.

Religion and science are conventionally polarised at two ends due to their classic epistemic and ontological divides. However, both subjects are unarguably developed and sustained by similar scholarly attitudes and share the same overarching goal of nurturing a critical mind—one that is open to argument, evidence and scepticism (Gauld, 2005). These commonalities of the two subjects have been disproportionately neglected in comparison to their disparities. Prolongedly driven by debates towards polarisation as well as unification, the two subjects are intrinsically the most interesting arena for researching argumentation and the question of knowing. Therefore, argumentation and its power in promoting cross-curricular learning can be effectively examined at the junction of religious and science education.

Argumentation is a pattern of reasoning (Basel et al., 2014). Toulmin’s (1958) framework of argumentation has been widely used to characterise the definition of argument (e.g. Bravo-Torija & Jiménez-Aleixandre, 2018). Toulmin presented an argument as having the following components: claim, data, warrant, backing, rebuttal, and qualifier. For the purposes of this paper, it suffices to say that an argument involves the justification (warrants and backings) of a claim with data under particular circumstances (qualifiers); and under certain conditions, a claim may be refuted (rebuttal). For example, Christians believe in the sanctity of life, that life is holy and belongs to God, and therefore, only God has the power to take life. Here, there is a claim that only God has the power to take life. The reason is that life is holy and it belongs to God. The justification being offered is the idea of the sanctity of life. This pattern of claim-reason-justification also applies to science. Plants require water to grow because water combines with carbon dioxide to produce oxygen and carbohydrates. In such case, the main claim is that plants require water to grow, and the reason is that water is part of a chemical reaction in plants. The justification of the argument is that growth is a chemical reaction. Some other claims might require putting scientific ideas and religious values together. For example, an issue such as why life originated on earth incites not only scientific but also religious curiosity. While the scientific arguments may draw on the biochemical mechanisms that enabled life to originate on the planet, the religious arguments might appeal to divine purpose in the creation of life. Although the arguments would be fairly distinct, they would be guided by the same question of why there is life at all.

Toulmin’s framework on argumentation addresses specific features of arguments in a particular domain (i.e. field dependent) as well as the generic aspects of argumentation that may apply to different domains (i.e. field invariant). For example, epistemic and methodological constructs can delineate and instantiate argumentation in teaching science, history, and literature (Goldman et al., 2018). These field-dependent rules specify what kind of information counts as evidence for a claim in science and how the criteria are different from those in history and literature. In short, argumentation encompasses a domain-general reasoning pattern based on evidence and reasons that operates across subjects and the epistemic nuances reflected in the particular justifications of different disciplines (Hetmanek et al., 2018; van Boxtel & van Drie, 2018).

Although argumentation has been extensively researched in various school subjects and in the context of socio-scientific issues (Martín-Gámez & Erduran, 2018) research about argumentation in RE is virtually non-existent (Erduran, 2020). Such lack of research is astonishing given that scientific and religious issues are often drawn together to be a focal axis in education research (Erduran et al., 2020). While argumentation research has thrived in science education over the last two decades, a blind spot emerged in RE. Nevertheless, a light within this blind spot is made dimly visible by a few scholars who studied argumentation in RE in German-speaking countries. Their interests in researching argumentation mostly concentrated on religio-scientific issues (Basel et al., 2013; 2014) or bioethical topics in schools (Schmidt et al., 2015; 2017), plus an outlier in pre-service teachers’ intercultural learning (Bender-Szymanski, 2013). Apart from this solitary group, other research on the English curriculum examined the role of RE in fostering generic intellectual skills which may entail argumentation (Chan et al., 2021).

It has been reported that teachers acknowledged the importance of teaching collaboratively to foster cross-curricular learning, but obstacles such as teachers’ limited expertise, roles, and policy in schools are not uncommon (Meskill & Oliveira, 2019; Spalding, 2002). Science teachers having to teach outside of their specialism at secondary school level is commonplace (Childs & McNicholl, 2007; Luft et al., 2020; Nixon et al., 2017). Due to the complex socio-political amalgam in teaching, science teachers often have to teach a scientific subject in which they have little background or limited subject-matter knowledge, for example, a biologist teaching physics. This systemic or institutional shortcoming negatively affects the development of science teacher’s pedagogical content knowledge and teaching in the classroom (McNicholl & Childs, 2010). Science teachers are also prone to isolation from collaborating with colleagues in other departments in school (Hargreaves, 1994; McNicholl & Childs, 2010; Spalding, 2002).

Much research on teachers learning about and teaching argumentation has focused on science education in schools. Some research has argued that teachers’ own experiences of learning science are limited to textbooks or exam syllabi and they have not learnt argumentation in their education trajectory (Sampson & Blanchard, 2012; Zembal-Saul & Vaishampayan, 2019). Since teaching argumentation is a higher-order skill which demands challenging one’s instructional practice in conceptual understanding as well as epistemic and social beliefs about learning (Zembal-Saul & Vaishampayan, 2019), it would not be prudent to rely on teachers to promote argumentation in classrooms if they themselves have no experience in engaging in scientific argumentation, or they are not supported to explore and understand this model and its potential for effective learning (Sampson & Blanchard, 2012; Zohar, 2007). Unsurprisingly, lack of pedagogical knowledge of teaching and supporting students in argumentation has been a common problem (Sampson & Blanchard, 2012). It is argued that conceptual strategies across subjects should be highlighted in teacher education so that teachers can be equipped to translate different cognitive models to other subjects (Cohen, 2018). Cross-subject collaboration among teachers in the same school is one strategy that can potentially enhance teachers’ pedagogical skills.

Although some research has explored science and RE teachers’ thinking about argumentation (Erduran et al., 2020; Guilfoyle et al., 2021), the teachers' views were given in a questionnaire when they were not collaborating with another subject teacher. The findings presented in this paper, which are based on semi-structured interviews, will thus augment this body of work by highlighting potential changes in teachers after a cross-subject collaboration and a continuous professional development (CPD) programme. The rest of the paper will hitherto investigate if and how such collaboration can be beneficial for science and RE teachers’ engagement with argumentation.

## Methodology

### Context and Participants

The data for this paper were taken from the *Oxford Argumentation in Religion and Science* (OARS), a funded research project on argumentation in science and RE classrooms in England (Erduran et al., 2019; Erduran et al., 2020; Godfrey & Erduran, 2021; Guilfoyle & Erduran, 2021). The teacher participants volunteered to take part after an invitation was circulated in local schools. Each science teacher was paired with an RE teacher in the same school to enable cross-subject collaboration. This paper presents three pairs of science and RE teachers from three secondary schools. Two of which were non-faith schools and the third was Catholic. All the six teachers were female and were chosen as examples of effective collaboration for reason of sample adequacy (Morse et al., 2002). Their teaching experience ranged from five to 32 years.

The project consisted of a CPD programme (see OARSeducation.com for further details). The programme was predicated on principles of effective professional learning such as teacher agency, shared goals and equity (Andrews & Richmond, 2019; Supovitz & Turner, 2000). These principles were embedded in five workshops over the course of 18 months. Research evidence suggests that novice science teachers are often caught up in classroom management with limited capacity to apply innovative strategies (Luft et al., 2011). In a similar vein, when experienced science teachers change schools or have to teach unfamiliar content, they resume the role of a novice science teacher (Loughran, 2007). To tackle these potential barriers, the CPD workshops presented work samples that would facilitate the participants to flexibly try out different strategies. Teachers explored argumentation within and between their subjects and sought to generate feasible ways of collaborating in their school. The ultimate goal of the collaboration was to develop students’ argumentation in science and RE. The co-learner model also encouraged the teachers to draw on and share their expertise with other teachers and the researchers. 

Teachers frequently work in silos (Hargreaves & O’Connor, 2018) so the CPD programme first guided the teachers to identify the common ground between science and RE through critically discussing the curriculam context. The second workshop examined the contrast of science and RE and designed lessons which focused on argumentation. For example, teachers explored the role of group discussion in developing argumentative skills. In the third workshop, teachers reflected on their school-based collaborations thus far. We observed that RE teachers were relatively comfortable in teaching argumentation, whereas science teachers expressed the need for more support. In fact, some RE teachers had a strong understanding of argumentation having completed doctoral degrees in fields such as philosophy and theology. Findings from the curriculum analyses were presented in workshop four, in which the teachers’ elaboration of the room for argumentation was also capitalised. The final workshop led teachers to review all the shared resources and reflect on how they embedded argumentation in school-wide activities. Over the 18 months, the teachers designed their collaboration and adapted it in the face of the pandemic.

Across the series of workshops, Toulmin’s framework of argument (Toulmin, 1958) was used to define argumentation. This framework has been utilised in science education (Lazarou & Erduran, 2021; Naylor et al., 2007; Zohar & Nemet, 2002). All teachers were interviewed at the start and end of their participation in this research. The interview questions sought to unpack the teachers’ views of argumentation, teaching argumentation, and their collaboration by the end of the project. For example, the teachers were asked how they had integrated argumentation in their lessons and what strategies had favoured their science-RE collaboration.

### Research Question

The study was guided by the following research question: *How does science and RE teachers' cross-subject collaboration impact their understanding and views of argumentation, teaching argumentation and their collaboration?*

### Data Collection and Analysis

The data set for our analysis is interview transcripts from three pairs of science and RE teachers. Understanding of argumentation, views about teaching argumentation, and collaboration were primarily adopted as three themes to examine the transcripts to ensure methodological coherence (Morse et al., 2002). This examination was then refined by highlighting any changes in those views and understanding or new perspectives to evidence CPD impacts. Both authors read the data separately, made notes, and then discussed together to reach shared interpretations, verification, and a confirmability audit (Nowell et al., 2017). The semantic analysis was facilitated by a constant comparison of the commonalities across the individual accounts (Parry, 2004; Polkinghorne, 2007). Given the flexibility of thematic analysis (Braun & Clarke, 2006), this iterative process was also enhanced by evaluating how the interviewees warranted their views and experiences (Silverman, 2017). Each discrete theme was first identified and then surveyed into an interrelated narrative guided by the research question to maintain consistency (Braun & Clarke, 2012). Emerging findings were also fed into the CPD workshops so that teachers could scrutinise those findings as part of our iterative analysis. This transparency was to make the research more meaningful to them.

## Findings

The findings are organised in three sections: (a) teachers’ understanding of argumentation, (b) teachers’ views of teaching argumentation, and (c) teachers’ views of collaboration. In each case, data from the three schools are used to illustrate science and RE teacher pairs’ understanding and views before and after the CPD intervention. In each section, data are drawn from the three schools labelled as A, B, and C, and the particular science or RE teacher is indicated.

### Teachers’ Understanding of Argumentation

The question “what is a good argument” gave us an introduction to what the teachers understood about argumentation. The initial responses by the RE and science teachers broadly aligned with the Toulmin’s model in the simplest manner. However, many of them thought engaging in argumentation demanded presenting contrastive views at the same time:

Compare, contrast, and come to conclusion… so that you can get practicing putting two things against each other. You’re not going to have a rigorous argument if one side is lacking. [B1/Sci]

Before the CPD intervention, the science teachers in the three schools emphasised the priority of learning scientific facts over practising argumentation, but this view was not mentioned by the RE teachers:

I think they [students] do have opinions. But they actually need enough information to be able to argue their point and back it up with facts. [A1/Sci]

After a series of CPD workshops and collaboration, the scientists became more aware of the need for critical thinking apart from linking “opinions” to “information” to formulate a claim. Evaluating a claim using evidence is an extra level in their understanding:

It is about developing students’ own view, being able to justify why they have that view. […] If they look at the evidence, do they need to rethink their opinion? In that way, they’re learning the information and they're growing. And discussing it, talking to each other. [A2/Sci]

The social aspect of learning had little place in the science teachers’ initial accounts in the three schools. “Talking to each other” was an evident shift from this science teacher’s pedagogical objective on scientific “information”. Her more articulate response about using “evidence” to “justify” students’ views aligned with Toulmin’s insertion of data and warrant to validate a claim. This reflective comment fathomed how the science teacher appropriated her understanding of Toulmin’s argumentation and connected it with the social dimension of arguing-to-learn.

Meanwhile, recognising the potentials of arguing-to-learn means the teachers in both subjects were able to appreciate the broader value of argumentation as a generic skill outside the classroom:

Actually, how argumentation tied into life skills, was one of the things that I did pick up on. [B2/Sci]

It just shows how argumentation as a skill. I guess it goes back to what [science teacher] said, it’s a life skill. [B2/RE]

Discussing argumentation also prompted the teachers to re-examine the nature of evidence in the two subjects and their convergence. The teachers illuminated what counts as evidence and explored its nuances between science and RE:

Different subjects might approach evidence from different ways. She’s [science teacher] seeing empirical knowledge only as evidence. I’m more open. I’m still trying to understand what is evidence in RE. [B2/RE]

Some of the things I wrote down was definitely explicit input on argumentation in the CPD sessions, which did help to clarify exactly what it was because I think when it comes to that warrant, argument and evidence thing, in the science aspects of it, we look at evidence slightly differently. [B2/Sci]

Here, the teacher pair was examining the field-dependent rules in argumentation (Toulmin, 1958), i.e. what qualifies as evidence in scientific reasoning and how it compares with evidence in RE. The nature of evidence categorically defines the disciplinary domain of the two subjects. Before working together on argumentation, the teachers delineated how science and RE differentiated in terms of what count as evidence and a claim. For example, the RE teacher shared her views on how the two subjects diverge, “science is all based in facts that are proved or data that is provided. RE is about interpretation of texts to make conclusions”. This divergence was agreed by her science colleague prior to their collaboration:

Claims in RE can be backed by evidence but it does not get you closer to a truth about the claim. You can contest claims with a great deal more philosophical thought that evidence from text may not be able to counter. I think RE is more about presenting evidence on both sides of an issue and then giving your own opinion. In science you hope that your evidence brings you further to finding an uncontested logical reasoned fact about the nature of the observed world. [C1/Sci]

Interestingly, at the end of their participation, both teachers agreed that on the basis of argumentation, the two subjects have more in common than they had thought:

But there was maybe a preconception on our part, that we were looking at very different types of writing and evidence, using evidence to develop an argument. When we dug deeper, we realise that no, we are the same. [C2/RE]

The same “types of writing and evidence” the RE teacher was referring to exemplify the domain-general rule in Toulmin’s argumentation (van Boxtel & van Drie, 2018). It is noteworthy that all the teachers’ post-intervention understanding of argumentation should not be interpreted as corrections of their pre-intervention views. Instead, we argue that their understanding became more sophisticated or complex. Through argumentation as a conceptual model, their understanding of RE and science might have taken a different angle, for instance, by examining what counts as evidence in a specific subject, but a large part of their understanding about the subject might remain unchanged. Engaging in argumentation has also led the teachers to rethink how to teach science by encouraging pupil talk.

## Teachers’ Views of Teaching Argumentation

Before the intervention, the science teachers lamented about the constraints of teaching science which made some of them hesitate about implementing argumentation:

The problem with science is that it's received knowledge and you don’t question it. There’s just so much factual stuff that you got to get into the little heads. […] This is constantly frustrating. [B1/Sci]

These comments on the science curriculum seem to relate to what the science teachers saw their role in teaching argumentation:

Students actually need enough information to be able to argue their point and back it up with facts. They need expert views, they need more than we know as classroom teachers. [A1/Sci]

This “expert views” showed a sharp turn after teaching collaboratively. Argumentative activity encourages students to explicate their scientific reasoning. This proactive process makes students’ potentials and difficulties visible. Students justifying and evaluating claims while organising the “factual stuff” prompted the teacher to assess understanding or misconceptions meaningfully:

It’s made me more aware of the fact that my kids might be thinking in other ways. So I've got to be more observant in the way in which they come to their conclusions. [B2/Sci]

This new avenue afforded by argumentation gave the science teachers confidence in adopting discursive pedagogy which engaged their students:

Sometimes I do allow room for discussions. I want them to find their voice in the whole knowledge being presented. I want them to become masters of their own views and of their own mind. [B2/Sci]

Bringing RE into science does make it more relevant to the students. It does help… to bring their own opinions rather than just me giving facts and saying, “this is how it is”. I really liked how most of the activities I did seem to be so accessible for all ranges of abilities. […] When we did the argumentation type lessons, they were involved in it. [A2/Sci]

The changes towards a student-centred perspective were more noticeable amongst the science teachers and not as much on their RE counterparts. Yet, the RE teachers also reflected on their increased understanding about students’ learning through coordinating with science:

I’ve learned that I didn’t actually have full knowledge of what the students were studying in science. [A2/RE]

This gain of knowledge about students learning argumentation was echoed by her science colleague:

I didn’t realise just how much science was being taught in RE. It’s been really thought-provoking, really getting me to think about the pupils’ experience in school. [A2/Sci]

All teachers in the three schools agreed that teaching argumentation in RE and science helped them to link different subjects together and learning would become integrated:

If you can draw in things from other disciplines, students realise that everything fits together. […] It had benefit to the students […] there is cross fertilisation between different disciplines. [B2/Sci]

It’s not just RE, not just science. Sometimes you bring something into the subject […] because RE pulls in lots of different things. [B2/RE]

Conjoining science and RE with other subjects would likely sharpen the teachers’ skills in cross-curricular learning which is a policy directive in many countries. Some teachers took the initiative to teach argumentation outside the target level and subjects of our research:

We now split into three different subjects. That’s an extra that they [students] were able to start those connections about how argumentation goes across the curriculum. [C2/RE]

Surprisingly, a few teachers also sustained teaching argumentation when coping with the challenges of teaching virtually due to Covid-19. Here is one example:

Back in lockdown, […] I literally had them in breakout rooms on Zoom, filling out one of these tables on literally point, evidence, explanation. [C2/RE]

Teaching argumentation in online classrooms was apparently beyond the teachers’ original intent when joining the study. Their adaptive experiences lend support to the versatile characteristics of argumentative activity reported in the literature.

## Teachers’ Views on Collaboration

Prior to this study, the duos mentioned that they had had no or little experience in collaborating with teachers from a different discipline or subject:

We’re in a small school so [before this research] I don't collaborate with anybody*.* [C1/Sci]

There’s no one like... I am on my own. [C1/RE]

In a collaborative partnership, the science or RE colleague became a useful resource which has enriched one’s own pedagogical repertoire:

I found it really, really interesting to look at how other subjects approach the topics, and how they set out their arguments. I did steal quite a few things from [RE teacher]. The collaboration was highly enjoyable. Because you get different perspectives. It makes life a lot more interesting. [RE teacher] and I disagree on quite a few things, quite robustly. But being able to have these conversations was lovely. [B2/Sci]

The professional exchanges brought fresh ideas and stimulated the teacher’s reverse thinking. Synchronising the focus on teaching argumentation in science and RE lessons has not only benefited student engagement but also the teachers’ pedagogical perspectives. The fruitful experiences motivated them to expand interdisciplinary collaboration in future:

I would definitely do it again. Probably with not just RE, but with some of my other colleagues, because we’ve got a lot of cross pollination between geography. […] That’s going to be a permanent change in insight, and how I think about what I need to do. [B2/Sci]

The successful collaboration affirmed the CPD programme principles which drew on teachers’ expertise to empower teachers’ agency in their pedagogical innovations:

This [teaching argumentation collaboratively] wasn’t something we were given to do. First of all we developed our own understanding, then we developed what it was that we were going to do so we owned that from the beginning. I think that also helped us with motivation. The fact that it was grown from us probably really helped it rather than anything else. [C2/RE]

All teacher participants enjoyed a high degree of pedagogical freedom to decide how they would collaborate. Over the course of the 18 months, no teachers were given a prescriptive scheme of work or materials to be replicated in any lessons. Neither was there any collaborative schedule to follow:

Maybe that was an advantage of all of it... it [the collaboration] became very specific to our needs; our school needs, our pupil needs, our learning needs. As opposed to kind of trying to shoehorn something you wanted into our school. [C2/RE]

The co-learner role adopted by the researchers also helped promote the sense of teacher ownership, which in turn increased the viability of implementing argumentation in different contexts.

## Discussion and Implications

Despite decades of research on argumentation particularly in science education (Erduran et al., 2015), Toulmin’s argument framework is still an unheard topic to many teachers. The three pairs of teachers featured in this paper were no exception. By comparing the pre- and post-intervention interviews, we have demonstrated that the six teachers reflected remarkable changes in how they understood argumentation following the CPD programme. These changes are especially profound in what they thought about the properties of an argument, the nuances of evidence, and the epistemic nature of the two subjects. Compared to the RE teachers, the scientists recounted more changes in their views about how argumentation prompts students to back up scientific ideas with evidence. Their changing views of argumentation are quite surprising, considering that teachers’ professional beliefs are generally stable or resistant to change (Clarke & Hollingsworth, 2002; Gregoire, 2003); and tensions may arise in shared pedagogical practice which requires teachers to cross subject boundaries (Aalton et al., 2019). Our findings offer insights into how understanding of argumentation is more than adopting a new conceptual framework such as Toulmin’s because it constitutes a teacher’s epistemological beliefs about the subject and what counts as knowledge in that subject boundary (Brownlee et al., 2017; Capps & Crawford, 2013; Strouper, 2014). Developing this understanding requires expertise on the nature of the subject as well as theory of knowledge at a meta level. Toulmin’s argument pattern provided the science teachers with a theoretical model to define what is science and to inspect if that contrasts with RE.

An overview of the findings from the three schools suggests that the teaching of argumentation created an epiphany to the science teachers to a larger extent than their RE counterparts. Prior to the CPD programme, RE teachers expressed greater engagement with pedagogical strategies related to argumentation than science teachers in a questionnaire (Erduran et al., 2020). Our findings from interviews corroborated the relative reservation shared by many science teachers, which concurrently explains their pronounced changes. Before the intervention, science teachers in different schools stressed the importance of teaching scientific facts and prioritised exam-oriented strategies over developing argumentation. They maintained a disciplinary focus and mentioned very little about how the two subjects might be related. These conceptions are unsurprising and are not limited to science. Curriculum overloaded with content knowledge may confine teachers to fixed subject boundaries and restrict them from facilitating students’ inquiry skills (Adolfsson, 2018). After a series of CPD activities and implementing argumentation in the classroom, the science teachers realised the potential of argumentation as a generic skill (Cohen, 2018) and an exam skill because evaluation of data is part of the science syllabus in high-stakes examinations (Childs & Baird, 2020). The teachers started to focus on students’ reasoning while instilling scientific facts, thus stating “I’m not reverting back to just giving the facts” by one of the science teachers. This revelation echoed the claim that the teacher is a facilitator and not an authority in teaching argumentation (Zohar, 2007). Understanding how teachers themselves engage with argumentation is paramount to its adoption in science education because “one must experience what it is like to come to understand the big ideas in science and the nature of science before s/he can be receptive to imagining the possibilities for designing opportunities for students’ science learning” (Zembal-Saul & Vaishampayan, 2019, p.168). Recent research has also shown that science teachers’ professional learning, their pedagogical content knowledge, and students’ understanding of scientific concepts are positively correlated (Yang et al., 2020).

The positive impact on the teachers’ views of teaching argumentation and collaboration is predominantly underpinned by the CPD programme designed for this study. The CPD programme was characterised by valuing teachers’ professional knowledge and judgement. This learning community consisting of teacher educators, researchers, and teachers was sustained by equity, inclusivity, and a shared goal of improving students’ thinking skills through interdisciplinary learning (Andrews & Richmond, 2019). Effective CPD programmes are usually those that are teacher-led, personalised, and congruent with teachers’ professional beliefs (Beisiegel et al., 2018; Noonan, 2019; Southerland et al., 2016). In this CPD context, the RE teachers were also a key professional resource for the science teachers, having reported using pedagogical strategies to support argumentation to a greater extent than the science teachers (Erduran et al., 2020). Before joining this research, all potential participants were well informed about the goals of the project, and the participation would involve co-teaching or co-planning lessons with a science or RE colleague. In the course of the research, the teachers were given considerable autonomy to collaborate in ways that would meet their specific needs. In each workshop, teachers were encouraged to share their different views or resources to exemplify a variety of collaborative styles and lesson designs. Interdisciplinary collaboration is growing in schools, and many policy initiatives have also urged for cross-curricular learning (OECD 2005; Ofsted, 2018). The experiences of collaborating with RE encouraged the science teachers to evaluate the relevance of argumentation to science education and the value of pupil talk. Future CPD promoting argumentation can be composed by teachers from various disciplines in one programme, for example, history teachers for their long advocacy of argumentation (Karras, 1993), and incorporate cross-curricular skills into the programme design.

By pairing up science and RE teachers and observing the outcomes in terms of changes in the teachers’ views, this paper contributes to the scarce literature that has involved both teacher cohorts in collaborative CPD. It sheds light on how science teachers’ learning can be supported through working with other subject teachers (Billingsley et al., 2020; Hardré et al., 2013). The teacher pairs identified an increasing intersection between science and RE, and such insight might have inspired their plan to collaborate with more subjects in the future. In this regard, the relational and contrary nature of the subject combination appears to be a distinctive feature (Edwards, 2017). Further professional development for science teachers should consider partnering with humanities or arts subjects to stimulate innovative pedagogical ideas or approaches.

Another contribution of this study is responding to the lack of argumentation research in RE. Although many RE curricular advocate an arguing-to-learn approach (Chan et al., 2021), argumentation in RE has been seriously under-researched. Future studies may explore its potential in RE in an array of faith and non-faith schools as well as curricular, though we argue that argumentation is a generic skill that is applicable to many subjects in different school contexts.

The paper raises questions about teachers’ learning when learning goals are shifted from learning subject/discipline knowledge to learning of the ways of reasoning in a subject, including “how claims are justified and validated, and what doing and engaging in discourse in the field entails” (Ball & McDiarmid, 1990, p. 438; Capps & Crawford, 2013). It is interesting that through discussing evidence, both science and RE teachers revisited argumentation as a life skill and discovered a bigger reconciliation between the two subjects. If the purpose of education is to develop learners who will function and engage effectively in democratic societies in meaningful ways as future citizens (Gutmann & Ben-Porath, 2015), then the teaching of argumentation can serve this important function (Erduran & Kaya, 2016).

Certainly, the six teachers in this paper do not represent many science and RE teachers outside our sample, yet our findings provide some insights into the generalisability of argumentation to cross-curricular learning, based on which the common ground between science and RE also becomes revealable (Gauld, 2005). Our descriptive findings are exploratory in nature because research on argumentation in RE has been empirically tested through this science-RE teacher collaboration (Swedberg, 2020). The teachers’ accounts by the science-RE pairs can inform future design of professional development, especially if it involves epistemic or interdisciplinary learning. A limitation of the study is the small teacher sample selected. Furthermore, the validity of those views would have been strengthened had they been verified by other research instruments. On this note, future research on CPD impact can focus on classroom practices in a cross-subject context.
